# LPS-induced modules of co-expressed genes in equine peripheral blood mononuclear cells

**DOI:** 10.1186/s12864-016-3390-y

**Published:** 2017-01-05

**Authors:** Alicja Pacholewska, Eliane Marti, Tosso Leeb, Vidhya Jagannathan, Vincent Gerber

**Affiliations:** 1 Department of Clinical Veterinary Medicine, Swiss Institute of Equine Medicine, Vetsuisse Faculty, University of Bern, and Agroscope, Länggassstrasse 124, 3012 Bern, Switzerland; 2 Department of Clinical Research and Veterinary Public Health, Institute of Genetics, Vetsuisse Faculty, University of Bern, Bremgartenstrasse 109A, 3012 Bern, Switzerland; 3 Department of Clinical Research and Veterinary Public Health, Division of Experimental Clinical Research, Vetsuisse Faculty, University of Bern, Länggassstrasse 124, 3012 Bern, Switzerland

**Keywords:** Lipopolysaccharides, LPS, Differential expression, Gene modules, WGCNA, Horse, RNA-seq

## Abstract

**Background:**

Lipopolysaccharide (endotoxin, LPS) is a strong inducer of the innate immune response. It is widespread in our environment, e.g. in house dust and contributes to asthma. Compared to humans, horses are even more sensitive to LPS. However, data on LPS effects on the equine transcriptome are very limited. Using RNA-seq we analysed LPS-induced differences in the gene expression in equine peripheral blood mononuclear cells at the gene and gene-network level in two half-sib families and one group of unrelated horses.

**Results:**

24 h-LPS challenge of equine immune cells resulted in substantial changes in the transcriptomic profile (1,265 differentially expressed genes) showing partial overlap with human data. One of the half-sib families showed a specific response different from the other two groups of horses. We also identified co-expressed gene modules that clearly differentiated 24 h-LPS- from non-stimulated samples. These modules consisted of 934 highly interconnected genes and included genes involved in the immune response (e.g. *IL6*, *CCL22*, *CXCL6, CXCL2*), however, none of the top ten hub genes of the modules have been annotated as responsive to LPS in gene ontology.

**Conclusions:**

Using weighted gene co-expression network analysis we identified ten co-expressed gene modules significantly regulated by *in vitro* stimulation with LPS. Apart from 47 genes (5%) all other genes highly interconnected within the most up- and down-regulated modules were also significantly differentially expressed (FDR < 0.05). The LPS-regulated module hub genes have not yet been described as having a role in the immune response to LPS (e.g. *VAT1* and *TTC25*).

**Electronic supplementary material:**

The online version of this article (doi:10.1186/s12864-016-3390-y) contains supplementary material, which is available to authorized users.

## Background

Innate immune response is the front line of the immune defence and therefore plays a crucial role for the organism’s survival starting at the time of birth. The innate immunity is non-specifically induced by invariant molecular structures present in pathogens, so called pathogen-associated molecular patterns (PAMPs) [1]. The best studied PAMP, lipopolysaccharide (LPS, also known as endotoxin due to its content of a toxic lipid A), is a component of the outer membrane of most gram-negative bacteria [2, 3].

When LPS enters the blood stream, most commonly through an intestinal lesion, it is opsonized by serum LPS-binding protein [4], which is recognized by Toll-like receptor 4 (TLR4) with the help of its co-receptor CD14 and another cell surface molecule, MD-2 [5, 6]. This recognition leads to a signalling cascade through MyD88- and TRIF-dependent pathways [7], activating the NF-κB transcription factor and finally inducing expression and release of numerous cytokines, including TNF-α, IL-1, IL-6, and IL-10 [8, 9]. This response and cascade are evolutionarily well conserved and thus very similar across species [10].

Sepsis is defined as a disrupted regulation of inflammation in the face of bacterial or other microbial infection, which can ultimately lead to tissue damage, organ failure, and death. This response may consist of the systemic inflammatory response (SIRS) or/and the compensatory anti-inflammatory response (CARS), and the balance between SIRS and CARS is crucial for the host survival [11, 12]. SIRS often results from infections caused by gram-negative bacteria [13, 14]. When sepsis and SIRS lead to clinically evident cardiovascular compromise, the result is termed septic shock [15], which is an uncontrolled life-threatening condition responsible for a large proportion of deaths of hospitalized patients worldwide [16]. Circulating LPS is believed to be the principal trigger of the septic shock [6].

In contrast to the above-described systemic effects of endotoxaemia (the presence of endotoxin in the blood), inhaled LPS can induce airflow obstruction and neutrophilic inflammation in healthy individuals. In asthmatic patients the effect of LPS may be either beneficial or harmful [17, 18], depending on the timing and dosage of the LPS exposure, as well as other environmental and genetic factors [17, 19–21]. In rats, early exposure to LPS, prior to sensitization with an allergen, can attenuate inflammatory processes in lungs, eosinophilia, and bronchial hypersensitivity, whereas endotoxin doses that are inhaled later on (6 days after allergen exposure) increase airway inflammation and edema [17, 22]. Low doses of LPS that induce a ‘normal’ Th1 type response may be beneficial as they are directing the Th1/Th2 balance towards Th1, thereby reducing the effects of the allergy related Th2 type response. However, it has also been shown that higher doses of LPS can contribute to occupational asthma [23]. The positive modulatory and negative exacerbating effects of LPS may be dependent on further microbial antigens and genetic effects [19, 24–26]. For instance, sequence variants in the *TLR4* gene can affect the responsiveness to LPS, and thereby influence the prevalence of asthma in a population [17, 19].

The degree of LPS sensitivity seems to be species-specific and horses are one of the most sensitive animals in their response to LPS exposure, while rodents appear to be much more resistant [11, 27, 28]. Endotoxaemia plays a major role in many equine diseases, particularly in intestinal disorders like acute colitis and ischemic bowel diseases, which often present with colic as the principal clinical manifestation [29]. Consequently, endotoxaemia is one of the main causes of mortality and morbidity in horses [30]. Horses suffering from colic have been shown to have increased LPS plasma concentrations and fatal colic cases had significantly higher LPS levels than non-fatal colic cases [31].

In equine recurrent airway obstruction (RAO, ‘equine asthma’), LPS does not cause the disease *per se*, which, similar to the pathophysiology of asthma, is due to a hypersensitivity to allergens in hay with a strong genetic basis [32–37]. However, hay dust can also contain high concentrations of LPS and inhaled endotoxin contributes to airway inflammation in RAO [38–40]. Furthermore, it is recognized that environmental exposure to microbial compounds, that do not result in clinical disease, but act through innate immune response mechanisms, influences the development of adaptive immunity and consequently allergy. Dendritic cells (DCs) are essential for priming T helper-2 differentiation of naïve T cells towards aeroallergens. However, contamination of antigens with PAMPs, such as LPS, is required to activate DCs to mount an immune response. Hammad *et al*. demonstrated in a mouse model of asthma that TLR4 triggering of epithelial cells, resulting in the release of innate pro-allergic cytokines, is necessary to drive allergic inflammation via activation of mucosal DCs [41]. To our knowledge equine global gene expression changes after LPS stimulation have not yet been analysed and reported. Genes function within networks that are typically redundant and regulatory mechanisms frequently assure that up- or down-regulation of a specific gene will be compensated by other genes [42]. It has been shown that studying groups of co-expressed genes, called gene modules, may better represent pathways of genes, which are co-regulated and/or interact with each other [43–47]. Weighted gene co-expression network analysis (WGCNA) is a tool that distinguishes modules of co-expressed genes by correlation and clustering analysis. The most highly connected genes within a module, ‘hub genes’, can then serve as good biomarkers that are characteristic for a phenotype studied, e.g. a disease.

Peripheral blood mononuclear cells (PBMCs) are a mix of several immune cell types circulating in the blood. They include cells that are involved in both the innate (monocytes, dendritic cells) and the acquired (lymphocytes) immune response systems. Therefore, PBMCs to a certain extent reflect the immune system status [48] and are widely used in systemic immune response studies [49–55].

Gene expression changes upon LPS challenge of immune cells have been intensively studied and well characterized in many mammalian species, but data in horses are limited despite the important impact of LPS both in equine gastrointestinal and respiratory diseases [29–31, 38–40]. Using a large RNA-seq dataset generated in the context of previous studies [50, 56], we report here the effect of 24 h in vitro LPS-stimulation on the transcriptome of equine PBMCs and the effect of genetic background on these LPS-induced transcriptomic changes.

## Methods

### Ethics statement

All animal experiments were performed according to the local regulations and with the consent of the horse owners. This study was approved by the Animal Experimentation Committee of the Canton of Bern, Switzerland (BE33/07, BE58/10 and BE10/13). The sample collection was previously described in detail in an earlier publication [49].

### Samples, RNA extraction and RNA-seq

This study is based on the results generated in a previously reported experiment and therefore all methods in detail are described elsewhere [49, 50, 56]. Briefly, blood samples were collected from 41 adult Warmblood horses (free of RAO) from three cohorts: two half-sibling families (Fam1: 7 horses, Fam2: 9 horses) and a group of unrelated horses (Un: 25 horses). About 8 million PBMCs from each horse were stimulated with LPS or left unstimulated (mock) for 24 h and subsequently frozen at -80 °C until RNA was extracted. High quality RNA (RIN > 8) was used for paired-end library preparation and sequenced on an Illumina HiSeq 2500 with 2 x 50 sequencing cycles. After quality control the sequencing reads were mapped to the horse reference genome (EquCab2). Raw data in binary-sequence alignment format (BAM) are available from European Nucleotide Archive (ENA) (http://www.ebi.ac.uk/ena/data/view/PRJEB7497) and Short Read Archive (SRA) (http://www.ncbi.nlm.nih.gov/Traces/sra/sra.cgi?study=ERP007230).

### Differential expression analysis

**Fig. 1:**
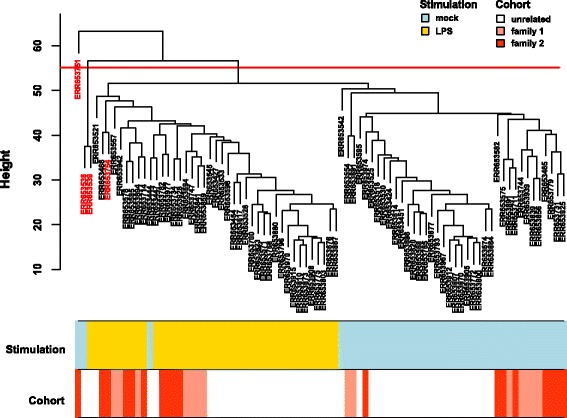
Sample hierarchical clustering. The samples clustered based on their Euclidean distance calculated with variance stabilized expression levels. The red line indicates the threshold line that excludes outliers from further analysis and excluded samples are marked in red in the dendrogram

Reads with mapping quality ≥ 20 were counted gene-wise with HTSeq [57] using the horse genome annotation from Ensembl (version 72) and default parameters. The differential expression analysis was performed with edgeR R package (Additional file 1) [58]. From the whole dataset used in our previous study [50, 56] we used 41 control PBMC samples that were cultured without any stimulating factor and 41 PBMC samples from the same horses stimulated with LPS and applied a filtering step in which we discarded all the genes with less than 10 counts in more than 90% of samples. As an initial quality control step we performed principal component analysis (PCA) using variance stabilized counts with individual horse effect removed using the removeBatchEffect function of limma R package (Additional file 1) [59, 60]. We next excluded outliers based on their Euclidean distances and visual inspection of the sample dendrogram (Fig. 1). Only horses with both mock and LPS stimulation were kept for further analysis (39 horses, 78 samples, Additional file 2).

For the model fitting, we applied a design that takes into account the effects of stimulation and cohort with interactions’ effects on the expression level:

$$ gene\  expression\sim stimulation+ cohort+ cohort: stimulation + cohort: horse $$

The unrelated group of horses was set as the reference group. The model was then fitted using the generalized linear model implemented in edgeR [58]. The effect of each factor on gene expression was tested and genes that were differentially expressed with a significance level of false discovery rate [61], FDR < 0.001 were assumed to be differentially expressed genes (DEGs). The stringent FDR threshold was set based on the large number of DEGs identified, please see Results for details.

The DEGs identified were compared with two other (microarray) studies available in public databases, where human PBMCs were stimulated with LPS for 24 h: [62, 63]. Using the GEO2R tool [64] we retrieved the DEGs between the control and 24 h-LPS stimulated human PBMCs. While many probes in a microarray can represent a single gene, we used only those genes, for which all probes were consistently significantly regulated by LPS with the same direction of regulation (up or down). From our list of DEGs we used genes with a gene symbol (genes without associated gene names were filtered out for this comparison as no human homologous gene could be identified). A stringent significance threshold of FDR < 0.001 was applied for enlisting the DEGs.

To get insight into the biological function of the DEGs, they were subjected to enrichment analyses with PANTHER overrepresentation test (release 20160715) [65–67] using the gene ontology (GO) biological process terms [68] and all 20,374 *Equus caballus* genes available from the PANTHER database as the reference set. Only the most specific GO subclasses from a group of all related parent classes in ontology were considered. The PANTHER default Bonferroni corrected *p*-value (*P*-value) significance threshold of 0.05 was used.

### Co-expressed network analysis

Signed co-expression networks were built using the WGCNA package in R [69] using variance stabilized counts with individual horse effect removed (Additional file 1) [59, 60]. BlockwiseModules function of the WGCNA package, which allows for network construction from the entire dataset, was used. For each set of genes a pair-wise correlation matrix was computed across the samples, and an adjacency matrix was calculated by raising the correlation matrix to the power of 12 using the scale-free topology criterion as suggested in [69].

Network interconnectedness (topological overlap measure) for each pair of the genes is calculated based on the adjacency matrix. The resulting topological overlap matrix is then converted to a dissimilarity measure and submitted to hierarchical clustering. The clustering produces a dendrogram, the branches of which represent similarly expressed genes, with the most highly connected nodal points or "hubs" located at the branch tips [69]. To cut the branches (cluster individual branches in separate "modules”), we used the hybrid dynamic tree-cutting because it leads to robustly defined modules [69]. We set the minimum module size to 40 genes and the minimum height for merging modules to 0.25 (Additional file 1).

Each module was summarized by the first principal component (i.e. eigengene) of the scaled (standardized) module expression profiles. The module eigengene is a single number that corresponds to the weighted average expression of all module genes in a sample [69]. For each module, the correlation between each gene expression values and module eigengene defines module membership (kME). The closer the absolute value of kME is to 1, the stronger the evidence that the gene belongs to the module represented by the module eigengene.

Significance of the LPS stimulation or cohort effect on the gene module was calculated using expression values of the module’s eigengene using limma package [60] with the same linear model as the one used for gene-wise differential expression analysis, without the horse effect removed before (Additional file 1). The genes initially assigned to the top two significant LPS-related modules and with high module membership (kME ≥ 0.7) were used for the enrichment analyses with PANTHER overrepresentation test [65] as described for DEGs.

## Results

### Differential expression analysis

**Fig. 2:**
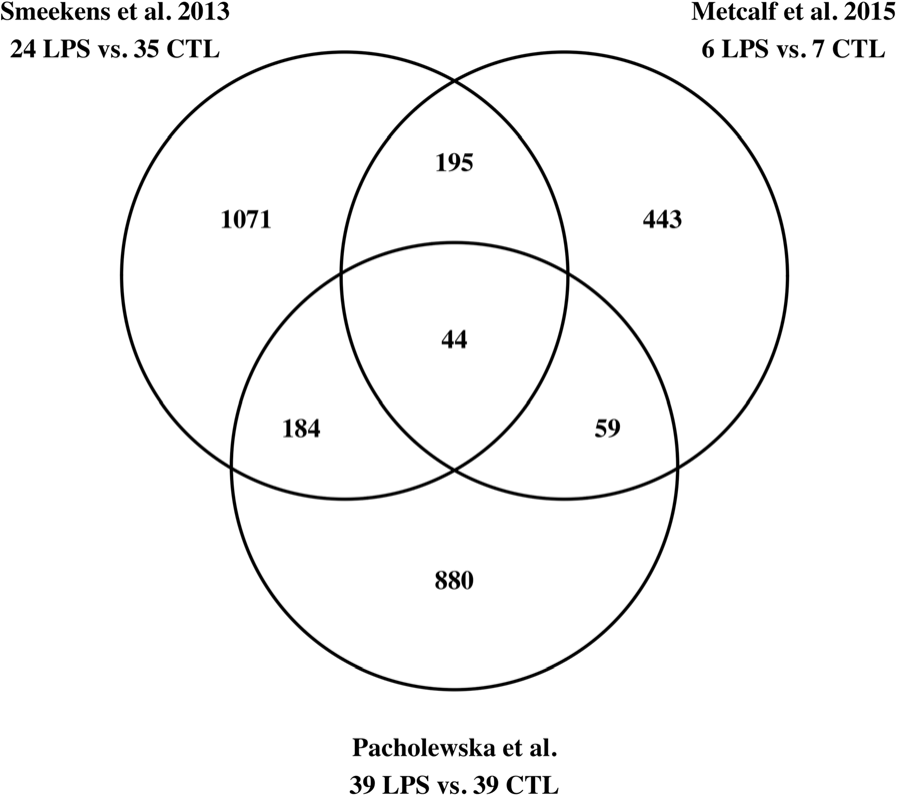
Number of common and study-specific DEGs. The venn diagram shows the overlap between the differentially expressed genes (DEGs) identified in equine (Pacholewska et al.) and human (Smeekens at al, Metcalf et al.) PBMCs stimulated with LPS for 24 h. From the equine gene list only genes with orthologous human gene symbols were examined. The number of replicates is denoted in the graph: LPS – samples stimulated with LPS, CTL – unstimulated control samples

We used a subset of a previously published RNA-seq dataset [50, 56]. Briefly, the 82 samples were derived from three different cohorts of healthy Warmblood horses: one group of horses that were unrelated at the parent level and two half-sibling families. The PCA showed a clear clustering of the samples according to the stimulation factor (Additional file 3). However, we removed three outliers based on the sample dendrogram (Fig. 1) plus one additional sample that matched the same horse as an outlier removed in order to keep only the samples with both mock and LPS stimulations required for the differential expression analysis. We identified 1,265 out of 12,855 analysed genes to be differentially expressed genes (DEGs) between LPS-stimulated and unstimulated samples in the reference group of 24 unrelated horses (FDR < 0.001, Additional file 4). The list of DEGs with associated gene names available (*n* = 1,167, 92% of all DEGs) partially overlapped DEGs from the other two studies on human PBMCs stimulated with LPS [62, 63]: 287 genes from the DEGs identified in this study were shared with at least one of the two other studies and 44 were shared by all three studies (Fig. 2). Log 2 fold changes (log2FC) of the DEGs identified in equine PBMCs ranged from -6.77 (insulin-like growth factor I, *IGF1*) to 4.81 (C-X-C motif chemokine 6 precursor, *CXCL6*).

**Fig. 3:**
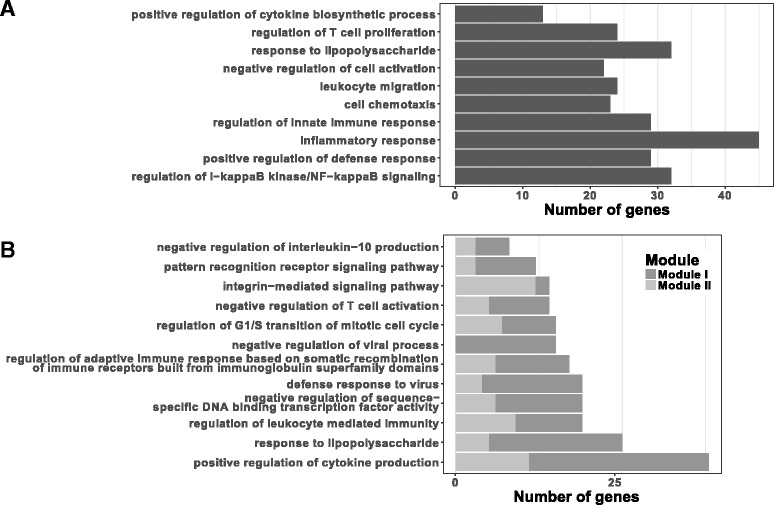
Number of DEGs annotated in the gene ontology (GO) biological processes. Most enriched and significant (Bonferroni corrected *p*-value < 0.05) biological processes annotated in the GO database and enriched by LPS-regulated genes identified in **a**) gene-wise and **b**) gene module-wise expression analysis (based on the GO database, release 2016-10-27 [65–67]). Each bar represents the number of genes enriched in the GO biological process. Bars are sorted by the fold enrichment with the most enriched processes on top

The PANTHER overrepresentation test [65] showed that the DEGs identified are involved in many immune response related processes (Fig. 3a). Ten most specific GO subclasses with the highest fold enrichments (FEs) are shown in Fig. 3a. The FE among the ten GO terms ranged from 4.69 to 2.81 (P-value range: 2.80e-6 – 4.67e-2). The only significantly enriched PANTHER pathway was the inflammation mediated by chemokine and cytokine signalling pathway (*P*-value = 6.10e-3; FE = 2.22). Of the 1,265 DEGs 37 genes (3%) were not matched to the PANTHER horse genome reference set of genes.

### Influence of the genetic background

We identified 16 genes (Additional file 4) that responded differently to stimulation with LPS in family 1 and 155 in family 2 compared to the unrelated cohort (Additional file 4, LPS:Fam1 and LPS:Fam2 effects). The LPS:Fam2 genes significantly enriched two GO biological processes: the nucleic acid metabolic process (32 genes, *P*-value = 1.86e-2), and cellular macromolecule metabolic processes (65 genes, *P*-value = 3.54e-6).

### Co-expression analysis

Normalized read counts per gene for the 78 samples grouped into two clusters accordingly to non- or LPS-stimulated group (Fig. 1). WGCNA identified 30 modules of co-expressed genes that consisted of 53 to 1,542 genes (Additional file 5). Only 636 genes (5%) were not included in any of the modules (by default WGCNA groups such genes into a dummy “grey” module). Every module was represented by its first principal component called the module eigengene (see methods for details). The association of eigengene expression with LPS stimulation and cohort was investigated using the linear model as explained in the Methods section. Of the 30 modules, ten were significantly associated with LPS stimulation in the reference group of 24 unrelated horses (FDR < 0.001), five modules were significant for the LPS:Fam2 interaction effect (module eigengenes responded differently to LPS stimulation in family 2 compared to unrelated cohort), and the most significant LPS:Fam2 module was also significant for the LPS:Fam1 effect (Additional file 6).

**Fig. 4:**
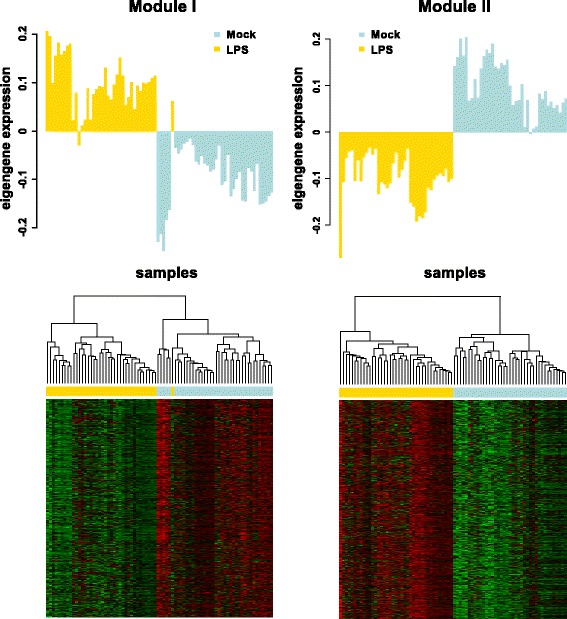
The two most significantly LPS-associated gene modules across samples. Bar plots represent the expression level of the module eigengenes across the samples. The light blue colour represents control mock-stimulated samples; the yellow colour represents samples stimulated with LPS. Heatmaps show the expression levels of all the module gene members with kME ≥ 0.7 (rows) across samples (columns). The expression values were scaled by rows and denoted by colour from red (low) to green (high). Dendrograms of samples were produced using hierarchical cluster analysis

The top significantly LPS-related modules (I and II) clearly divided the samples into two separated clusters of LPS-stimulated or unstimulated cells as shown in Fig. 4. The barplots represent the expression of the module eigengene and heatmaps the expression of the module genes (kME ≥ 0.7) across the samples. The genes with the strongest correlation (membership) with the top two LPS-related modules’ eigengenes are listed in Table 1.

**Table 1:** The genes with the strongest membership with the top two LPS-related modules

Module	Module hub genes	Module membership	LPS log2FC	DE FDR
module I	*BIRC3*	0.97	1.26	1.89e-12
module I	*VPS37A*	0.95	0.53	3.97e-9
module I	*STARD13*	0.94	0.80	1.01e-4
module I	*SOD2*	0.94	1.75	8.83e-16
module I	*SLC1A2*	0.94	2.83	3.62e-18
module I	*ARHGEF3*	0.94	0.53	3.39e-8
module I	*TTC25*	0.94	0.84	5.30e-8
module I	*SLC39A14*	0.93	1.80	2.80e-12
module I	*ENSECAG00000002023,* superoxide dismutase	0.93	1.79	5.69e-16
module I	*TREX1*	0.93	0.85	2.25e-7
module II	*VAT1*	0.95	−1.13	1.68e-16
module II	*SDC3*	0.94	−2.27	1.16e-9
module II	*FCGRT*	0.94	−1.39	1.42e-10
module II	*SLC46A1*	0.94	−1.80	8.21e-18
module II	*NAAA*	0.94	−0.94	2.53e-20
module II	*CSF1R*	0.93	−1.54	1.43e-11
module II	*MAN2B1*	0.93	−0.44	5.77e-10
module II	*ARRB1*	0.92	−1.03	3.19e-9
module II	*GAMT*	0.92	−2.40	3.77e-19
module II	*S100A4*	0.92	−1.10	4.29e-12

For every module the ten most correlated genes are listed with the following features: module membership, log2 fold change of the expression regulated by LPS, false discovery rate for the gene-wise differential expression (DE)

The I and II module genes (with kME ≥ 0.7) were further subjected to the enrichment analysis. We retrieved all 12 significant GO biological processes with FE ≥ 2.81 (FE ≥ of the tenth most enriched biological process by the LPS-induced DEGs). FE among the 12 GO terms ranged from 12.74 to 3.11 (P-value range: 1.27e-5 – 3.1e-2). The processes were mostly related to the immune response and included the response to LPS, as well as a cell cycle related process (Fig. 3b). The most enriched PANTHER GO-Slim biological process was the negative regulation of apoptotic process (*P*-value = 4.72e-2), and none of the PANTHER pathway was significantly enriched. Of the 934 module I and II genes 20 (2%) were not matched to the PANTHER horse genome reference set of genes.

## Discussion

LPS is known to be a strong inducer of the innate immune response and changes in the gene expression can be observed within one hour post LPS challenge [3, 70]. Actually, the majority of the studies on the LPS-induced immune response focus on short-term effects. In contrast, this study investigated the consequences of a 24 h LPS-challenge, which may better reflect continued exposure to LPS, which is the cause of increased morbidity and mortality in equine gastrointestinal [29–31] and respiratory diseases [38–40]. As expected, in the equine PBMCs studied here, 24 h LPS stimulation affected the regulation of a substantial number of genes, almost 10% of all genes studied at a FDR < 0.001. This fraction increased to more than 24% of the studied genes when a less stringent FDR threshold of < 0.05 was applied. The magnitude of this effect was in accordance with previous reports using genome-wide expression analyses demonstrating that LPS strongly affects the transcriptomic profile of blood cells revealed by in vitro and in vivo studies in humans [62, 63, 71–74].

While the horse is more sensitive to LPS than other mammals that have been studied (including humans) [27], the innate immune response to LPS is very similar across species. This includes specific cell types and mediators involved in the inflammatory cascade following LPS challenge and is likely due to the fact that innate immunity is a highly conserved, evolutionarily old immune response [10].

Interestingly, our results from the differential expression analysis agreed in part with similar studies on human LPS-stimulated PBMCs [62, 63] (Fig. 2). Almost a quarter of DEGs identified in our study overlapped DEGs from both the studies on human PBMCs. It has to be also taken into account that the overlap between the two human studies was also surprisingly small: only 32% of DEGs identified by Metcalf et al. was identified in the study of Smeekens et al.; and only 15% vice versa. From a total of 239 common DEGs between the two studies on human PBMCs, 18% were also identified in our study. While these two studies focused on the defence against *Candida albicans* and the age effect on innate response, they employed the same duration of 24 h-LPS-stimulation as in our study [62, 63]. Moreover, many of the equine LPS-regulated genes (8%) did not have human homologs and therefore had to be excluded from this comparison.

Although the immunological reaction to LPS has already been described in several species, the response to LPS is still poorly investigated in horses with only 41 unique equine proteins listed under the GO term ‘response to LPS’ (GO: 0032496, state of 23rd September 2016) with evidence based on computational analysis. Of these 41 protein-coding genes 18 were also supported by our study at FDR < 0.05 (*CD40, CXCL2, CXCL6, CXCL8, F2R, IL18, MIP-2BETA, PPBP, TLR4, TNFRSF11B, TNFRSF18, TNFRSF1B, TNFRSF21, TNFRSF4, TNFRSF8, ENSECAG00000012397, ENSECAG00000012560, ENSECAG00000015037*) and 11 when the stringent threshold was applied (FDR < 0.001). The most up-regulated gene in our dataset, the chemokine *CXCL6*, attracts neutrophils that are known to be involved in the first line of the immune response [75, 76]. Among the up-regulated DEGs, there were also genes previously identified as LPS-induced genes in humans at the mRNA (using reverse transcriptase quantitative PCR) or protein level (e.g. *IL1B*, *IL6*, *IL8 (*also known as *CXCL8), CCL22*, *CXCL6*) [75].

Previous genome-wide transcriptome profiling of whole blood samples derived from paediatric patients with SIRS, sepsis, or septic shock revealed putative use of circulating IL27 in the blood as a biomarker for sepsis in human patients [77, 78]. Interestingly, in our study both genes encoding for the two subunits of IL27 (*IL27* and *EBI3*) were up-regulated in LPS stimulated cells.

It is worth mentioning that sepsis is characterized by a ‘cytokine storm’ in the first phase, followed by a second phase, after approximately 24 h, where the immune response is ‘paralysed’ due to the apoptosis of activated lymphocytes [79–81]. The suppression of the immune response increases the risk of secondary infections in patients with sepsis, which in turn represent a major cause of death in these patients [82]. The apoptosis during immune paralysis in sepsis is believed to be induced by caspase signals, namely death receptor induced caspase 8 (*CASP8*) or mitochondrial induced caspase 9 (*CASP9*) [83, 84]. Although we did not identify significant changes in the expression levels of *CASP8* or *CASP9*, the most markedly down-regulated gene by the LPS stimulation was the insulin-like growth factor 1 gene (*IGF1*). IGF1 has been shown to have an anti-apoptotic effect [85–88]. This decrease was in accordance with another study in which LPS was shown to suppress the expression of IGF1 in post mortem derived human microglia on both mRNA and protein levels [89].

Because many genes’ functions are still unknown and because genes often work within complex networks, we hypothesized that the analyses of co-expressed groups of genes could facilitate a deeper insight into LPS-induced immune responses. Therefore, we assigned genes to modules based on their co-expression. Ten out of 30 modules identified possessed significant relationships with LPS stimulation.

Interestingly, our analysis revealed that horse family 2 is more distant from the unrelated group in their response to LPS than family 1, based on both differential expression and co-expression analysis (Additional files 4 and 6). We noticed earlier that occurrence of equine asthma (RAO) is correlated with an increased resistance against parasites in unrelated Warmblood horses and in family 1, but not in family 2 [49, 50, 90, 91]. Hub genes of the three LPS:Fam2 specific modules that were not significant for LPS:Fam1 may thus play a role in the response to parasites.

As expected, some of the LPS-related modules identified in this study confirmed previous findings in regards to our knowledge of LPS-induced immune responses, but our analysis also revealed interesting novel aspects. Genes of the two most strongly LPS-related modules showed clear differences in the expression in stimulated versus unstimulated PBMCs, as shown in Fig. 4. Among the top significant LPS-related module genes there were genes involved in immune response, including the response to LPS (Fig. 3b). Indeed, the WGCNA analysis revealed 272 genes from the two modules not identified as LPS-regulated based on gene-wise differential expression analysis at FDR < 0.001. Only 47 of these were not DEGs at the more permissive FDR threshold of 0.05.

Although module I included one of the hallmark-genes of LPS-stimulation, *IL6,* the hub genes in this module were *BIRC3*, encoding the baculoviral IAP repeat containing 3, and *VPS37A*, encoding vacuolar protein sorting 37 homolog A. *BIRC3* was already implicated in the pro-inflammatory cytokine induction [92] and *VPS37A* in the inhibition of viral infection [93]. The third hub gene was StAR related lipid transfer domain containing 13 (*STARD13*), which is believed to supress cell proliferation and motility [94] and therefore may be important for the regulation of the immune response upon stimulation with LPS.

Even more interesting are hub genes from the LPS-downregulated module: albeit the function of the vesicle amine transport 1 protein encoded by *VAT1* gene has not been fully described, it has been shown that this gene is expressed in human neutrophils and VAT1 protein is located peripherally in unstimulated neutrophils with a more granular pattern upon stimulation with a macrophage activator [95, 96]. Since our collection of PBMCs did not contain neutrophils, the function of VAT1 in PBMCs requires further investigation.

Surprisingly, we identified up- and down-regulated genes involved in both positive and negative regulation of apoptosis, e.g. the anti-apoptotic *IGF1* gene, which was the most down-regulated DEG in LPS-stimulated samples. Apart from *IGF1* module I hub genes included guanidinoacetate N-gethyltransferase gene (*GAMT*) that was also decreased in LPS-samples. This gene may have also an anti-apoptotic role as it has been reported that fibroblast cell lines from two GAMT-deficient patients showed increased production of mitochondrial reactive oxygen species and apoptotic rate [97]. As apoptosis induces immunosuppression [98], the reduced expression of the anti-apoptotic genes (*IGF1*, *GAMT*) may have a role in the immune suppression phase of sepsis and support the concept of LPS induced immune regulation/paralysis.

Another indication of the immune regulation by long-term stimulation with LPS in our study was the down-regulation of another hub gene, syndecan 3 (*SDC3*, module I). SDC3 serves as a synovial binding site for IL-8 on endothelial cells [99] thus down-regulation of *SDC3* results in a decrease in binding sites for the inflammatory IL8. The immune regulation/paralysis could be further supported by the down-regulation of the N-acylethanolamine acid amidase (*NAAA*) and Fc fragment of IgG receptor and transporter gene (*FCGRT*), module II hub genes. NAAA degrades N-acylethanolamines in macrophages and is involved in inflammatory processes [100] and has previously been reported as potential anti-inflammatory agent [101–104]. FCGRT protects immunoglobulin G from degradation, thus its expression reduced may be the result of the immune response regulatory mechanisms [105].

What is noteworthy is that none of the 20 hub genes of the I and II modules listed in Table 1 has been annotated in the GO term response to lipopolysaccharides (GO:0032496). Among these hub genes was also the tetratricopeptide repeat domain 25 gene (*TTC25*) that has been poorly characterized so far. Nonetheless, this gene seems to play an important biological role in the respiratory tract and other organ systems that depend on intact ciliary function, as it has been recently reported that mutations in the *TTC25* lead to primary ciliary dyskinesia [106]. However, we also need to caution that this study was performed on RNA-seq data only and individual gene specific function and their role in LPS-related pathways would still require further functional analysis.

## Conclusions

The WGCNA revealed novel aspects in an already much studied field, such as the response to LPS. We identified two co-expressed gene modules that clearly separated LPS- from non-stimulated cells. Apart from known LPS-response genes (e.g. *IL6*, *IL8*, *CXCL6*) the two modules identified comprised novel genes with potential roles in LPS-response pathways (e.g. *VAT1* and *TTC25*). Moreover, the regulation of genes involved in the apoptotic process in our data suggests that 24 h stimulation with LPS may reflect the ‘immune paralysis’ phase observed in patients with bacterial sepsis. In addition, we further confirmed that the family 2 horses differ in their immune response from family 1 and unrelated horses.

## Additional files

Additional file 1: Script used for the data analysis. An R script used for the gene-wise differential expression analysis with edgeR [58] and weighted gene co-expression network analysis with WGCNA R package [69]. (PDF 38 kb)
Additional file 2: Data used for the analysis in R. An RData file that contains compressed objects required for the differential expression and co-expression analyses with the script from Additional file 1. It contains the count and design tables. (RDATA 2007 kb)
Additional file 3: Principal component analysis. Principal components were calculated on variance stabilized counts before and after implementing the removeBatchEffect function of limma R package [59, 60]. All 82 samples were plotted across two first principal components and coloured according to the stimulation (blue – unstimulated; yellow – stimulated with LPS). (PDF 42 kb)
Additional file 4: Lists of DEGs between LPS stimulated and unstimulated PBMCs. For every effect studied (LPS, and interaction effects: LPS:Fam1, LPS:Fam2) the list of genes differentially expressed at false discovery rate < 0.001 with log 2 fold changes is given. (XLSX 198 kb)
Additional file 5: Dendrogram of genes assigned to the modules. The colours in the bar below the dendrogram represent the modules. The grey colour represents genes not assigned to any of the modules. The dendrogram was obtained by average linkage hierarchical clustering [69]. (PDF 255 kb)
Additional file 6: Co-expressed gene modules. For every module the list of genes with module membership, and FDR values of the module association with LPS stimulation and cohort is given. (XLSX 6286 kb)

## Abbreviations

CARS: Compensatory anti-inflammatory response
DC: Dendritic cell
DEG: Differentially expressed gene
FDR: False discovery rate
FE: Fold enrichment
GO: Gene ontology
kME: Module membership
Log2FC: Log 2 fold change
LPS: Lipopolysaccharides
PAMP: Pathogen associated molecular pattern
PBMC: Peripheral blood mononuclear cell
PCA: Principal component analysis
RAO: Recurrent airway obstruction (horse asthma)
SIRS: Systemic inflammatory response
WGCNA: Weighted gene co-expression network analysis.
