# Supplementary material for: LPS-induced modules of co-expressed genes in equine peripheral blood mononuclear cells
Source: BMC Genomics. 2017 Jan 5;18:34. doi: 10.1186/s12864-016-3390-y (PMC5217269; doi:10.1186/s12864-016-3390-y)
Supplement: Additional file 1: — Script used for the data analysis. An R script used for the gene-wise differential expression analysis with edgeR [58] and weighted gene co-expression network analysis with WGCNA R package [69]. (PDF 38 kb) [file 12864_2016_3390_MOESM1_ESM.pdf]

```
#####################################################################
#-------------------------------------------------------------------#
# Gene- and gene module-wise differential expression analysis      #
# of equine PBMCs stimulated with LPS                               #
#-------------------------------------------------------------------#
#####################################################################
# Last modified: 17.11.2016
# Author: Alicja Pacholewska & Vidhya Jagannathan
# Article: "LPS-induced modules of co-expressed genes in equine
#          peripheral blood mononuclear cells"
# Contact: alicja@rth.dk; vidhya.jagannathan@vetsuisse.unibe.ch
#
# Binary sequence alignment/map (bam) files are available here:
# http://www.ebi.ac.uk/ena/data/view/PRJEB7497
#
#-------------------------------------------------------------------#
# FILES DESCRIPTION:
# annot   - EquCab2 gene associated names from Biomart v. 84
# counts  - table with raw counts from 78 samples (39 control horses
#           with both Mock and LPS stimulation) collected with HTSeq
#           genes with <10 counts in >90% samples) removed
# LPSsubj - design table with phenotypic data
#
# ABBREVIATIONS:
# LPS - lipopolysaccharides
# CTL - control

#-------------------------------------------------------------------#
# INSTALLING NECESSARY R PACKAGES
#source("http://bioconductor.org/biocLite.R")
#biocLite (pkgs=c("edgeR","DESeq2","WGCNA","flashClust",
                 "RColorBrewer","gplots"))

#Load necessary files:
load("Additional_file_2.RData")

#Make sure that count table columns correspond to LPSsubj rows
counts<-counts[,rownames(LPSsubj)]
dim(counts)

#Make sure annotations match gene counts
table (rownames(annot) %in% rownames(counts))

#Load necessary packages
library("edgeR")
library("DESeq2")
library("WGCNA")
library("flashClust")
library("RColorBrewer")
library("gplots")

# Create a color palette for heatmaps
my_palette <- colorRampPalette(c("brown","red", "black", "green",
                                 "darkgreen"))(n = 299)
```

```
#-------------------------------------------------------------------#
#                      DE analysis with edgeR                       #
#-------------------------------------------------------------------#

#assign samples to single groups
group<-factor(paste(LPSsubj$stimulation, LPSsubj$cohort,
                    LPSsubj$horse, sep = "_"))

#Create DGE object with edgeR
dge<-DGEList(counts = counts, group = group)

#Normalize the counts
dge<-calcNormFactors(dge)

#Use model that accounts for individual horse effect
des<-model.matrix(~stimulation + cohort + cohort:stimulation +
                      cohort:horse, data = LPSsubj)
#remove empty columns/factors
keep<-apply(des, 2, max)
des<-des[,keep > 0]

#Estimate dispersions
dge<- estimateDisp(dge, des)

#fit the model
fit <- glmFit(dge, des)


#-------------------------------------------------------------------#
# Counts normalization and multidimensional scaling analysis

# Normalize the raw counts
vst <-varianceStabilizingTransformation(dge$counts)

# Remove individual horse effect
data <-removeBatchEffect(vst,
                         design = des[,-grep("horse",colnames(des))],
                         batch = LPSsubj$horse)

#-------------------------------------------------------------------#
#Estimate the LPS effect and the LPS:Fam1 and LPS:Fam2 interaction
effects:

LPS<-glmLRT(fit, coef = 2)
LPSFam1<-glmLRT(fit, coef = 5)
LPSFam2<-glmLRT(fit, coef = 6)

#The effects are closely related, we use global p value correction
FDR<-
p.adjust(c(LPS$table$PValue,LPSFam1$table$PValue,LPSFam2$table$PValue),
method = "fdr")

FDRFam1<-FDR[(length(FDR)/3+1):(length(FDR)/3*2)]
FDRFam2<-FDR[(length(FDR)/3*2+1):length(FDR)]
```

```
FDR<-FDR[1:(length(FDR)/3)]

#Select significant DEGs for LPS effect
x<-cbind(gene_name = annot[rownames(LPS$table,),], LPS$table, FDR)
#x<-x[FDR<0.05,]
x<-x[FDR<0.001,]
LPS<-x[order(x$FDR),]
#Save the results
write.table(LPS, file = "LPSeffect.txt",sep = "\t", quote = FALSE)

#Save significant DEGs for LPS:Fam1 effect
x<-cbind(gene_name = annot[rownames(LPSFam1$table),], LPSFam1$table,
FDRFam1)
#Select significant DEGs
#x<-x[FDRFam1<0.05,]
x<-x[FDRFam1<0.001,]
LPSFam1<-x[order(x$FDRFam1),]
write.table(LPSFam1, file = "LPSFam1effect.txt", sep ="\t",quote =
FALSE)

#Save significant DEGs for LPS:Fam2 effect
x<-cbind(gene_name = annot[rownames(LPSFam2$table),], LPSFam2$table,
FDRFam2)
#Select significant DEGs
#x<-x[FDRFam2<0.05,]
x<-x[FDRFam2<0.001,]
LPSFam2<-x[order(x$FDRFam2),]
write.table(LPSFam2, file = "LPSFam2effect.txt", sep="\t", quote =
FALSE)

#------------------------------------------------------------------#
#      Weghted gene co-expression network analysis with WGCNA      #
#------------------------------------------------------------------#

#Choose the parameters:
nType = "signed"
cutoff = 0.25 #cutoff for module merging
FDR_t = 0.001
pow = 12 # soft threshold

# data frame is now transformed to contain samples as rows and genes
# as columns
datExpr<-as.data.frame(t(data))

#The following setting is important, do not omit.
options(stringsAsFactors = FALSE)

#check the data
gsg = goodSamplesGenes(datExpr, verbose = 3)
gsg$allOK

if(!gsg$allOK)
{ if (sum(!gsg$goodGenes) > 0) printFlush(
  paste("Removing genes: ",paste(
    names(datExpr)[!gsg$goodGenes], collapse = ", ")))
```

```
  if(sum(!gsg$goodSamples) > 0) printFlush(
    paste(" Removing samples:",
          paste(rownames(datExpr)[!gsg$goodSamples],
                collapse =", ")))
  datExpr = datExpr[gsg$goodSamples, gsg$goodGenes]
}


#The variable datExpr now contains the expression data ready for
# network analysis.
nGenes = ncol(datExpr)
nSamples = nrow(datExpr)

# Loading the LPSsubj Traits and convert the factors to numeric
# (for the graphics to work)
Samples<-rownames(datExpr)
traitRows<-match(Samples,rownames(LPSsubj))
datTraits<-LPSsubj[traitRows,]
datTraits<-datTraits[,2:3]
datTraits$cohort<-as.numeric(as.factor(datTraits$cohort))
datTraits$stimulation<-as.numeric(as.factor(datTraits$stimulation))

#We now have the expression data in the variable datExpr, and
#the corresponding clinical traits in the variable datTraits.
#visualize how the clinical traits relate to the sample dendrogram.
# Cluster samples
sampleTree = flashClust(dist(datExpr), method = "average")
## Convert traits to a color representation
traitColors = numbers2colors(datTraits, signed = FALSE);
# Change stimulation colors to light blue and gold
traitColors[,1]<-gsub("#FFFFFF", "#ADD8E6", traitColors[,1])
traitColors[,1]<-gsub("#FF3300", "#FFD700", traitColors[,1])

# Plot the sample dendrogram and the colors underneath.
pdf(file="sampleClusteringheatmap_withTraits.pdf",width=12,height=9)
sizeGrWindow(12,9)
par(cex = 0.6)
par(mar =c(0,4,2,0))
plotDendroAndColors(
  sampleTree, traitColors,
  groupLabels = names(datTraits),
  main ="Sample dendrogram and trait heatmap",)
dev.off()

#------------------------------------------------------------------#
#Choosing the soft-thresholding power: analysis of network topology

#the power was set to 12 based on the following analysis:

##Choose a set of soft-thresholding powers
#powers =c(c(1:10),seq(from = 12, to = 20, by = 2))

## Call the network topology analysis function
#sft = pickSoftThreshold(datExpr, powerVector = powers, verbose = 5,
#                         networkType = nType)
```

```
## Plot the results of soft thresholding:
#pdf(file="softThreshold.pdf",width = 9, height = 5)
#sizeGrWindow(9, 5)
#par(mfrow = c(1,2))
# Scale-free topology fit index as a function of the soft-
# thresholding power
#plot(sft$fitIndices[,1], -sign(sft$fitIndices[,3])*
#       sft$fitIndices[,2], xlab = "Soft Threshold (power)",
#     ylab = "Scale Free Topology Model Fit,signed R^2", type="n",
#     main = paste("Scale independence"));
#text(sft$fitIndices[,1], -sign(sft$fitIndices[,3])*
#       sft$fitIndices[,2], labels = powers, cex = 0.7, col ="red");
#abline(h=0.7,col = "red")
## Mean connectivity as a function of the soft-thresholding power
#plot(sft$fitIndices[,1], sft$fitIndices[,5],
#     xlab = "Soft Threshold (power)",ylab = "Mean Connectivity",
#     type = "n",main = paste("Mean connectivity"))
#text(sft$fitIndices[,1], sft$fitIndices[,5], labels = powers,
#     cex=0.7, col = "red")
#dev.off()

#-------------------------------------------------------------------#
# Identify gene modules

datExpr<-apply(datExpr, c(1,2), as.numeric)
net = blockwiseModules(datExpr, power = pow, maxBlockSize = nGenes,
                       networkType=nType, minModuleSize = 40,
                       reassignThreshold= 0, mergeCutHeight = cutoff,
                       numericLabels = FALSE,
                       pamRespectsDendro = FALSE,
                       saveTOMs = FALSE,verbose = 3)

#Summarize the gene modules
table(net$colors)
#the grey module collects all the remaining genes not included in any
#of the true modules

#Calculate module eigengene values (MEs) and plot ME relationships
moduleLabels = net$colors;
moduleColors = net$colors;

# Module eigengene values (MEs)
corMx=cor(net$MEs, use="p")
#Clustering dendrograms of consensus module eigengenes for
#identifying meta-modules.
cluster = hclust(as.dist(1-corMx), method = "a")
pdf("plotClusterMEsLPSSTIM.pdf", height = 10, width = 10)
plot(cluster, main="Module Eigengene Dendrogram")
dev.off()
# pairwise scatterplots of eigengenes
pdf("plotMEpairsLPSSTIM.pdf", height = 40, width = 40);
plotMEpairs(net$MEs)
dev.off()
```

```
# Convert the numeric labels to color labels
moduleColors = labels2colors(moduleLabels)

# Plot gene dendrogram with the assignment to a module
consTree = net$dendrograms[[1]];

sizeGrWindow(8,6);
pdf(file = "ConsensusDendrogram-auto.pdf", wi = 8, he = 6)
plotDendroAndColors(consTree, moduleColors,
                    "Module colors",
                    dendroLabels = FALSE, hang = 0.03,
                    addGuide = TRUE, guideHang = 0.05,
                    main = "Consensus gene dendrogram
                    and module colors")
dev.off()

#Collect Module Eigengene values
meInfo<-data.frame(net$MEs)
rownames(meInfo)<-rownames(datExpr)

#Reassign the modules and get the module membership
KMEs<-signedKME(datExpr, net$MEs,outputColumnName = "kME")
#remove grey module with unassigned genes
KMEs<-KMEs[,-which(colnames(KMEs)=="kMEgrey")]

#-------------------------------------------------------------------#
# Relate the modlues to LPS stimulation and cohort
options(stringsAsFactors = TRUE)

des2<-des[Samples,1:6] #remove samples removed before from the design

#ignore the horse effect that has been removed
fit<-lmFit(t(meInfo), des2)
fit <- eBayes(fit)


#LPS effect
topTable(fit,coef = 2)
LPS<-topTable(fit, coef = 2, n=length(levels(factor(net$colors))))
LPSFam1<-topTable(fit, coef = 5, n=length(levels(factor(net$colors))))
LPSFam2<-topTable(fit, coef = 6, n=length(levels(factor(net$colors))))

FDR<-p.adjust(
  c(LPS$P.Value, LPSFam1$P.Value, LPSFam2$P.Value), method = "fdr")

LPS$adj.P.Val<-FDR[1:(length(FDR)/3)]
LPSFam1$adj.P.Val<-FDR[(length(FDR)/3+1):(length(FDR)/3*2)]
LPSFam2$adj.P.Val<-FDR[(length(FDR)/3*2+1):(length(FDR))]

data<-cbind(LPS,LPSFam1[rownames(LPS),],LPSFam2[rownames(LPS),])
data<-data[,grep("adj",colnames(data))]
colnames(data)<-c("LPSeffect","LPSFam1effect","LPSFam2effect")
data<-data[-(grep("MEgrey$",rownames(data))),]
write.table(data, file="modules.txt",
```

```
            quote = FALSE, sep = "\t")


#-------------------------------------------------------------------#
# Plot eigengene/module genes expression for the two most LPS-
# related modules

for(I in 1:2){

  #Plot originally assigned module genes heatmap
  which.module=gsub("ME", "", rownames(topTable(fit, coef = 2))[I])
  d<-datExpr[,which(net$colors==which.module)]
  d<-as.matrix(t(d))
  colo<-factor(LPSsubj[rownames(datExpr),2], levels=c("Mock","LPS"))
  levels(colo)<-c("lightblue", "gold")
  pdf(paste(which.module, "heatmap.pdf", sep = "_"))
  a<-heatmap.2(d, scale="row",trace="none",
               labCol=LPSsubj[colnames(d),3], # plot sample names
               #labCol = rep("",dim(d)[2]), # remove sample names
               cexCol = 0.5,labRow=rep("", dim(d)[1]),
               col = my_palette,key=FALSE,dendrogram="column",
               ColSideColors = as.character(colo))
  dev.off()

  #Plot the expression of module eigengene across samples
  pdf(paste(which.module, "Eigengene.pdf", sep = ""));
  barplot(net$MEs[a$colInd,rownames(topTable(fit, coef = 2))[I]],
          col = as.character(colo)[a$colInd], main="", cex.main = 2,
          ylab ="eigengene expression",xlab="samples", font.lab = 2,
          font.axis = 2, border = NA)
  legend("topright",legend=c("Mock","LPS"),
         fill = as.character(levels(colo)), border=NA, bty = "n")
  dev.off()

  #Write genes for enrichment analysis (take only genes with strong
  #module membership)
  k<-as.data.frame(cbind(gene_name=as.character(annot[rownames(d),]),
                         kME=KMEs[rownames(d),
                                  paste("kME",which.module,sep="")]))
  #write originally assigned genes with module membership
  rownames(k)<-rownames(d)
  k$kME<-as.numeric(as.character(k$kME))
  write.table(k[k$kME >= 0.7 ,],
              file = paste(which.module, "_genes.txt", sep = ""),
              quote = FALSE, sep="\t")
}

#Write module membership for each gene and for each module.
KMEs<-KMEs[,paste("k",rownames(data),sep="")]
geneInfo=data.frame(annot[rownames(KMEs),1],moduleColors,KMEs)
colnames(geneInfo)[1:2]= c("gene_name",
                           "Initially Assigned Module Color")

#Save kME table and MEs
write.csv(geneInfo, "geneInfoCTLSTIM.csv", quote=FALSE)
```

```
write.csv(meInfo, "meInfoCTLSTIM.csv", quote=FALSE)

sessionInfo()

R version 3.2.3 (2015-12-10)
Platform: x86_64-apple-darwin13.4.0 (64-bit)
Running under: OS X 10.11.4 (El Capitan)

locale:
  [1] C

attached base packages:
  [1] parallel  stats4    stats     graphics  grDevices utils
datasets
[8] methods   base

other attached packages:
  [1] gplots_3.0.1              RColorBrewer_1.1-2
[3] flashClust_1.01-2         WGCNA_1.51
[5] RSQLite_1.0.0             DBI_0.5-1
[7] fastcluster_1.1.21        dynamicTreeCut_1.63-1
[9] DESeq2_1.10.1             RcppArmadillo_0.7.500.0.0
[11] Rcpp_0.12.7               SummarizedExperiment_1.0.2
[13] Biobase_2.30.0            GenomicRanges_1.22.4
[15] GenomeInfoDb_1.6.3        IRanges_2.4.8
[17] S4Vectors_0.8.11          BiocGenerics_0.16.1
[19] edgeR_3.12.1              limma_3.26.9

loaded via a namespace (and not attached):
  [1] locfit_1.5-9.1        lattice_0.20-34      GO.db_3.2.2
[4] gtools_3.5.0          assertthat_0.1       digest_0.6.10
[7] foreach_1.4.3         plyr_1.8.4           chron_2.3-47
[10] futile.options_1.0.0  acepack_1.4.1        ggplot2_2.2.0
[13] zlibbioc_1.16.0       lazyeval_0.2.0       gdata_2.17.0
[16] data.table_1.9.6      annotate_1.48.0      rpart_4.1-10
[19] Matrix_1.2-7.1        preprocessCore_1.32.0 splines_3.2.3
[22] BiocParallel_1.4.3    geneplotter_1.48.0   stringr_1.1.0
[25] foreign_0.8-67        munsell_0.4.3        compiler_3.2.3
[28] htmltools_0.3.5       nnet_7.3-12          tibble_1.2
[31] gridExtra_2.2.1       htmlTable_1.7        Hmisc_4.0-0
[34] codetools_0.2-15      matrixStats_0.51.0   XML_3.98-1.5
[37] bitops_1.0-6          grid_3.2.3           xtable_1.8-2
[40] gtable_0.2.0          magrittr_1.5         scales_0.4.1
[43] KernSmooth_2.23-15    impute_1.44.0        stringi_1.1.2
[46] XVector_0.10.0        genefilter_1.52.1    doParallel_1.0.10
[49] latticeExtra_0.6-28   futile.logger_1.4.3  Formula_1.2-1
[52] lambda.r_1.1.9        iterators_1.0.8      tools_3.2.3
[55] survival_2.40-1       AnnotationDbi_1.32.3 colorspace_1.3-0
[58] cluster_2.0.5         caTools_1.17.1       knitr_1.15
```
