# Supplementary figures and images for: LPS-induced modules of co-expressed genes in equine peripheral blood mononuclear cells
Source: BMC Genomics. 2017 Jan 5;18:34. doi: 10.1186/s12864-016-3390-y (PMC5217269; doi:10.1186/s12864-016-3390-y)

**VST normalized counts**

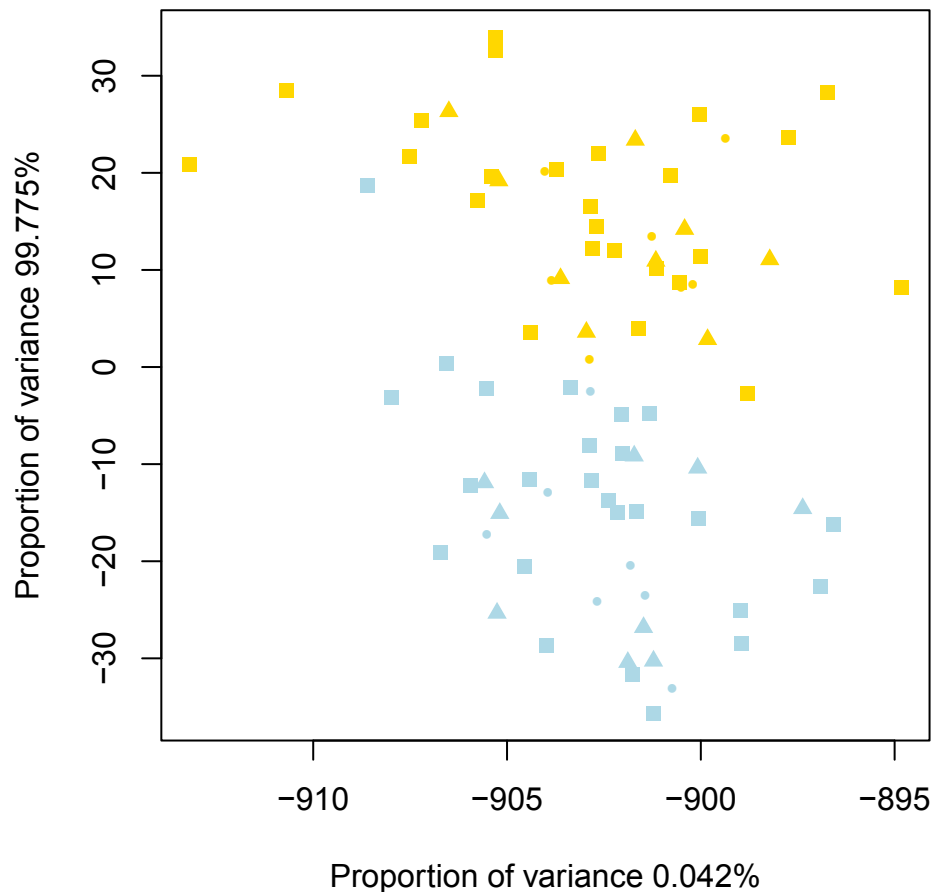

**VST normalized counts with the horse effect removed**

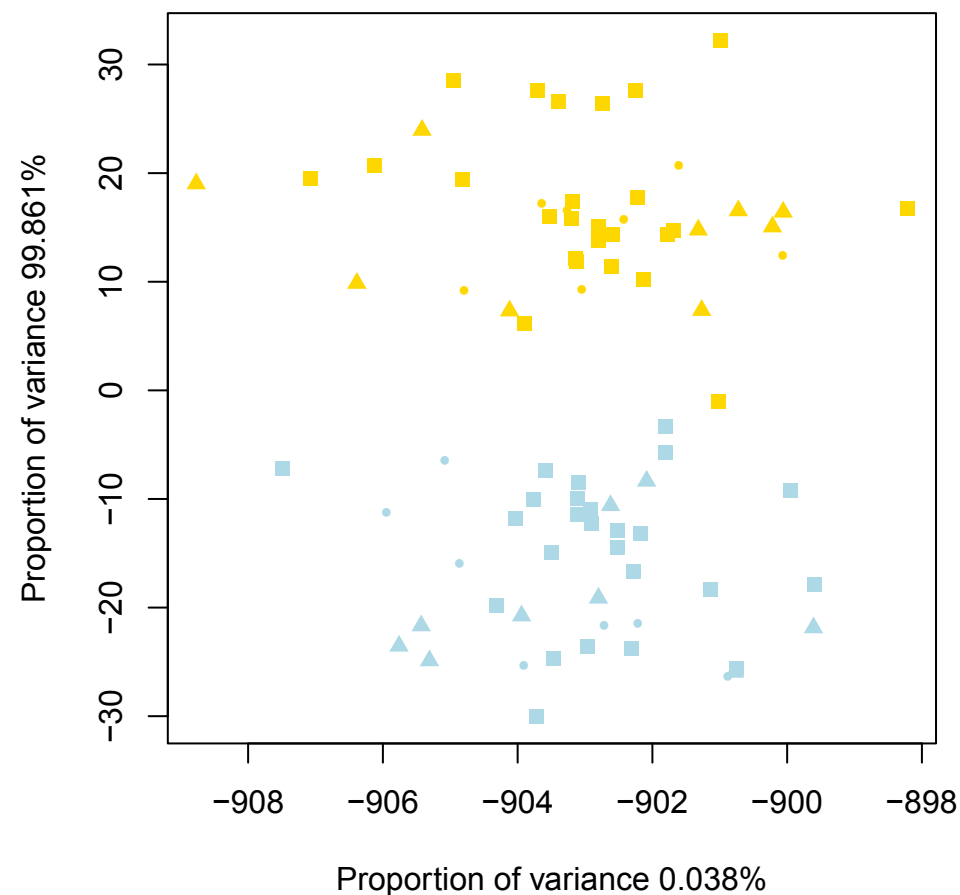

Supplement: Additional file 3: — Principal component analysis. Principal components were calculated on variance stabilized counts before and after implementing the removeBatchEffect function of limma R package [59, 60]. All 82 samples were plotted across two first principal components and coloured according to the stimulation (blue – unstimulated; yellow – stimulated with LPS). (PDF 42 kb) [file 12864_2016_3390_MOESM3_ESM.pdf]

# Consensus gene dendrogram and module colors

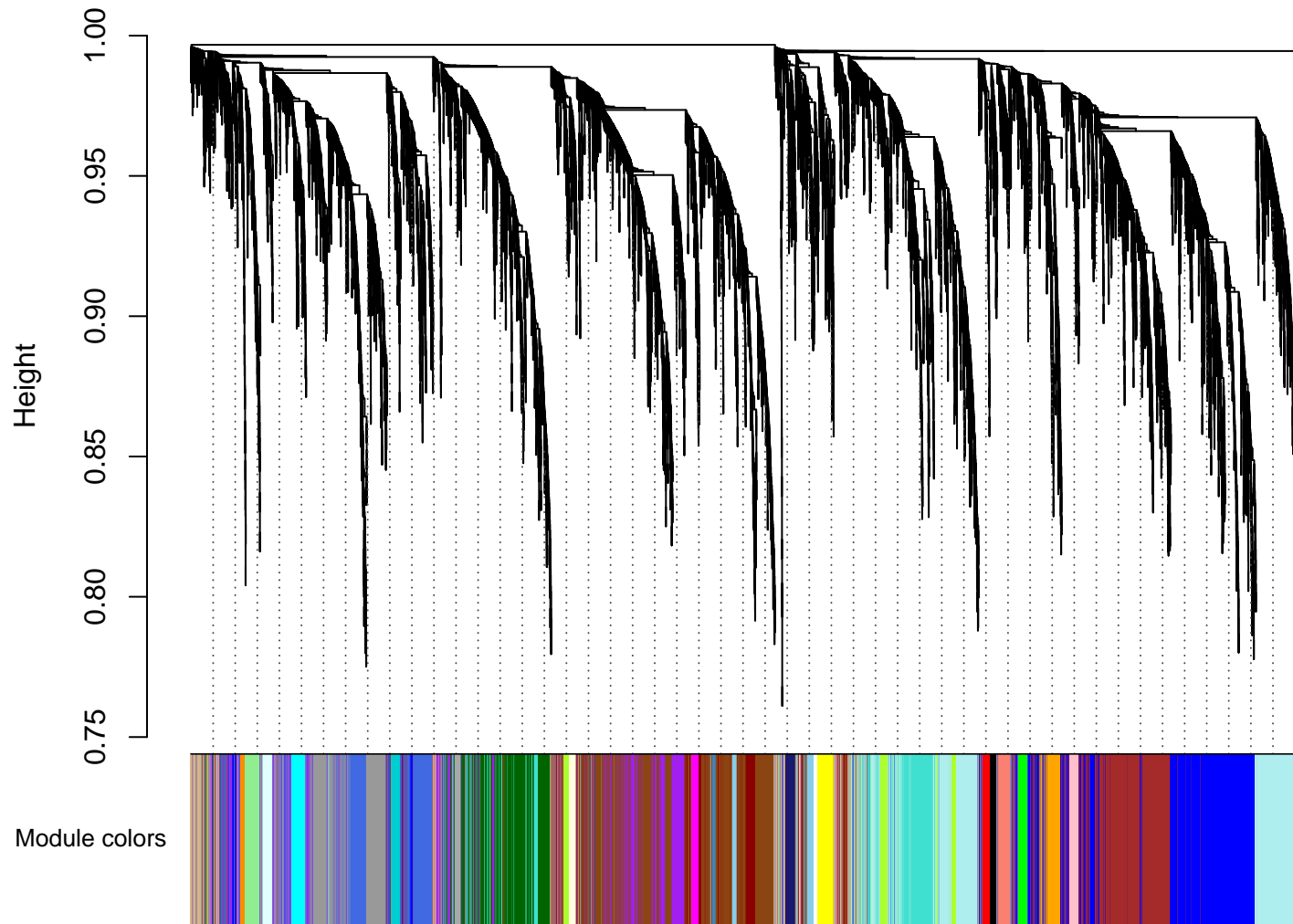

Supplement: Additional file 5: — Dendrogram of genes assigned to the modules. The colours in the bar below the dendrogram represent the modules. The grey colour represents genes not assigned to any of the modules. The dendrogram was obtained by average linkage hierarchical clustering [69]. (PDF 255 kb) [file 12864_2016_3390_MOESM5_ESM.pdf]
